# National Mass Drug Administration Costs for Lymphatic Filariasis Elimination

**DOI:** 10.1371/journal.pntd.0000067

**Published:** 2007-10-31

**Authors:** Ann S. Goldman, Victoria H. Guisinger, Moses Aikins, Maria Lourdes E. Amarillo, Vicente Y. Belizario, Bertha Garshong, John Gyapong, Conrad Kabali, Hussein A. Kamal, Sanjat Kanjilal, Dominique Kyelem, Jefrey Lizardo, Mwele Malecela, Godfrey Mubyazi, P. Abdoulaye Nitièma, Reda M. R. Ramzy, Thomas G. Streit, Aaron Wallace, Molly A. Brady, Richard Rheingans, Eric A. Ottesen, Anne C. Haddix

**Affiliations:** 1 Department of Epidemiology and Biostatistics, The George Washington School of Public Health and Health Services, Washington, DC, United States of America; 2 Lymphatic Filariasis Support Center, Emory University, Atlanta, Georgia, United States of America; 3 Health Research Unit, Ghana Health Service, Accra, Ghana; 4 Department of Clinical Epidemiology, College of Medicine, University of the Philippines, Manila, The Philippines; 5 Department of Parasitology, College of Public Health, University of the Philippines, Manila, The Philippines; 6 National Institute of Medical Research, Dar es Salaam, Tanzania; 7 Ministry of Health & Population, Cairo, Egypt; 8 Lymphatic Filariasis Program, Ministry of Health, Ouagadougou, Burkina Faso; 9 Center for Social Management, Technological Institute of Santo Domingo, Santo Domingo, Dominican Republic; 10 Department of Evaluation, Ministry of Health, Ouagadougou, Burkina Faso; 11 Research & Training Center on Vectors of Diseases, Ain Shams University, Cairo, Egypt; 12 Department of Biological Sciences, University of Notre Dame, Notre Dame, Indiana, United States of America; Case Western Reserve University School of Medicine, United States of America

## Abstract

**Background:**

Because lymphatic filariasis (LF) elimination efforts are hampered by a dearth of economic information about the cost of mass drug administration (MDA) programs (using either albendazole with diethylcarbamazine [DEC] or albendazole with ivermectin), a multicenter study was undertaken to determine the costs of MDA programs to interrupt transmission of infection with LF. Such results are particularly important because LF programs have the necessary diagnostic and treatment tools to eliminate the disease as a public health problem globally, and already by 2006, the Global Programme to Eliminate LF had initiated treatment programs covering over 400 million of the 1.3 billion people at risk.

**Methodology/Principal Findings:**

To obtain annual costs to carry out the MDA strategy, researchers from seven countries developed and followed a common cost analysis protocol designed to estimate 1) the total annual cost of the LF program, 2) the average cost per person treated, and 3) the relative contributions of the endemic countries and the external partners. Costs per person treated ranged from $0.06 to $2.23. Principal reasons for the variation were 1) the age (newness) of the MDA program, 2) the use of volunteers, and 3) the size of the population treated. Substantial contributions by governments were documented – generally 60%–90% of program operation costs, excluding costs of donated medications.

**Conclusions/Significance:**

MDA for LF elimination is comparatively inexpensive in relation to most other public health programs. Governments and communities make the predominant financial contributions to actual MDA implementation, not counting the cost of the drugs themselves. The results highlight the impact of the use of volunteers on program costs and provide specific cost data for 7 different countries that can be used as a basis both for modifying current programs and for developing new ones.

## Author Summary

Lymphatic filariasis (LF), commonly known as elephantiasis, is a profoundly disfiguring parasitic disease caused by thread-like nematode worms. This disease can often be disabling, thus reducing the potential productivity of the affected individuals. The WHO places the number of people at risk in 83 countries at 1.307 billion. This study was undertaken in seven countries—Burkina Faso, Ghana, Egypt, Tanzania, the Philippines, the Dominican Republic, and Haiti—using a common protocol to determine the costs of mass drug administration (MDA) programs to interrupt transmission of infection with LF, because there is lack of sufficient information about the costs of these programs. The results demonstrate that LF MDA is affordable and relatively inexpensive when compared to other public health programs. In the context of initiatives for integrating programs for the control and elimination of neglected tropical diseases, this study adds specifically to the relatively scarce body of information about the costs of MDA programs for LF. It also adds to the general knowledge about the application of methods that can be used to estimate the costs and cost-effectiveness of an integrated approach.

## Introduction

Lymphatic filariasis (LF), commonly known as elephantiasis, is a profoundly disfiguring parasitic disease caused by thread-like nematode worms. The World Health Organization (WHO) places the number of people at risk in 83 countries at 1.307 billion.[1] Globally, the reduced productivity as a consequence of LF disability has been well recognized. The chronic and debilitating burden of LF maintains the cycle of poverty not only in infected individuals but also in entire endemic communities. [2,[3] Indeed, as a disease of poverty, LF is endemic in 43 of the 50 countries classified as least developed nations. [4–6]

The 1997 World Health Assembly resolved to eliminate LF as a public health problem after LF had been identified as one of only a small number of diseases classified as potentially eradicable. [7] The principal strategy for elimination relies on once-yearly concurrent administration of two drugs, albendazole with diethylcarbamazine (DEC) or albendazole with ivermectin – both regimens shown to be highly effective in removing microfilariae from the blood for a full year after treatment. [8] Administration of these once-yearly, single-dose regimens to people in at-risk communities for 4–6 years makes feasible the prospect of interrupting transmission and thereby eliminating LF, [9] largely because the reproductive life span of the adult worm is estimated to be 4–6 years. While the delivery of DEC fortified salt is another strategy that has been applied in some regions of the world to eliminate LF, this strategy was not in the purview of the current studies.

Demonstrating that LF elimination is a cost-effective and affordable investment is essential for both Ministries of Health and potential donors as they choose among competing health needs. While several cost-effectiveness analyses have been conducted and one estimate for MDA in high prevalence areas gives a range of $4–8 per disability adjusted life year (DALY) averted, [2, 10–13] more cost and cost-effectiveness data are lacking. The persistent need for detailed costing of mass drug administration (MDA) programs led to the present initiative to develop and implement a common cost-analysis protocol. Countries participating in this multi-country study were selected to represent the different stages and scope of national LF elimination efforts, different economic conditions of endemic countries, and different geographic regions affected by the disease. Health expenditures per capita in the participating countries for 2001–2002 ranged from $12 in Tanzania and Ghana, to $22 in Haiti, $27 in Burkina Faso, $30 in the Philippines, $46 in Egypt and $153 in the Dominican Republic.[14,15] The cost analysis objectives were to 1) estimate total annual costs of the LF program for a specific year or more (depending on the availability of information), 2) calculate the average cost per person treated, 3) identify the relative contributions of the endemic country and external partners, 4) provide data that could be used for program evaluation and analysis, 5) understand how the results differed among countries, and 6) build a framework for the development and implementation of cost studies elsewhere.

Cost analyses aggregated in this report are based on studies in Burkina Faso, the Dominican Republic, Egypt, Ghana, Haiti, the Philippines, and Tanzania. Detailed analyses from two of these countries have already been published elsewhere.[12,16]

## Methods

### Study Teams

Data collection and analysis were carried out by research teams in each of the participating countries. The cost analysis teams in Burkina Faso, Tanzania, and Ghana were part of the Ministry of Health. In Egypt the study was a joint effort between the Ministry of Health and Population and the Ain Shams University Research and Training Center on Vectors of Diseases. The Philippines' research team was based at the National Institutes of Health-Philippines. The Dominican Republic researchers at the Centro de Gerencia Social, Instituto Tecnologico de Santo Domingo worked with the Ministry of Health LF program. External consultants in Haiti worked with Ministry of Health LF program staff to collect and analyze cost study data.

### Study Protocol

Researchers from the participating countries collaborated with the Emory University LF Support Center in the development of a protocol (http://www.taskforce.org/lfsc/professionals.html) which served as the tool to identify, organize, analyze, summarize and present the cost data. The protocol and accompanying data collection instrument created a systematic process for data collection and analysis so that the cost estimates would be comparable across a variety of country settings, programmatic approaches and program sizes.

The cost analysis identified both *economic costs* (*i.e.,* the value of *all resources* used in the program, including donated items, such as medications for the MDA) and *financial costs* (*i.e.,* the *actual cash disbursements* for a program including resources provided by the national government and local communities). Economic costs are useful to evaluate the allocation of program resources and their opportunity costs, e.g., whether these resources could be used more productively elsewhere. Financial costs are helpful to program managers to measure actual program expenditures and assess affordability.[17] (See Table 1 for the classification of costs.) Financial costs include all costs with the exception of donated materials. Capital items were annualized to reflect costs incurred in one year for the project. They do not include bicycles because these were the property of the volunteer drug distributors, and thus considered a donation. Volunteer training per diems are classified as Financial and Economic costs, but the value of volunteer unpaid time is not included in either Financial or Economic costs. Because the capital costs were annualized, the cash expenditures described reflect those incurred in one year for the project.

**Table 1:** Classification of Costs.

Inputs	Economic Costs	Financial Costs
Albendazole	*yes*	*no*
Bicycles	*yes*	*no*
Clinic space	*yes*	*yes*
Computers	*yes*	*yes*
DEC	*yes*	*yes*
Donated materials	*yes*	*no*
Existing vehicle	*yes*	*yes*
Food and refreshments	*yes*	*yes*
Fuel and maintenance	*yes*	*yes*
Mectizan®	*yes*	*no*
Office Space -MOH	*yes*	*yes*
Office Space -rented	*yes*	*yes*
Office Supplies	*yes*	*yes*
Other drugs	*yes*	*yes*
Printing	*yes*	*yes*
Purchased vehicle	*yes*	*yes*
Staff time	*yes*	*yes*
Utilities	*yes*	*yes*
Volunteer training per diem	*yes*	*yes*
Volunteer time	*no*	*no*

The protocol adopted a national program perspective because most resources dedicated to the LF MDAs were channeled through Ministry of Health (MOH) programs. Participants agreed to gather MDA costs, beginning with the year 2000, the first year of the LF global elimination effort. Costs were calculated in local currencies and converted to US dollars for the final analysis, based on average exchange values for the years being analyzed (base year 2002).

Most of the countries were still in the early stages of expanding their MDAs when the cost analysis studies began. The exception was Egypt where the MDA targeted almost 90% of the population at risk the first year and 100% the second year studied.

Project inputs defined in the protocol were: personnel, supplies and drugs (medications for the MDA and for treatment of adverse reactions), as well as capital and recurrent costs for equipment, transportation, and facilities. Each input was allocated to one or more categories of activities involved in accomplishing the MDA: training, mapping, mobilization and education, drug distribution, adverse reaction monitoring, surveillance/laboratory, and administration (Table 2). Capital costs were defined as one-time investments in physical goods that have a life longer than one year and generally cost more than $1000. These costs were annualized, using a formula that accounted for years of useful life, scrap value, and a discount rate set at 3%. Recurrent costs included those items that were consumed on a regular basis; i.e., personnel time, office and laboratory supplies, fuel, and drugs. Recurrent costs for maintenance of donated capital items such as bicycles were also included. In cases where facilities or equipment costs were not available, costs of similar facilities or rentals were used as a proxy. For the purpose of Economic costs, the value of the donated drugs was set as $0.19 plus $0.0019 for shipping per 400mg tablet of albendazole (Personal Communication: GlaxoSmithKline. February 17, 2004) and $1.50 plus $.0018 per 3 mg tablet of Mectizan (Personal Communication: Merck & Co., Inc. March 1, 2004) DEC, used in four of the seven countries studied, was not donated by the private sector and had to be purchased for the national programs.

**Table 2:** MDA Activities and Other Cost Categories.

Activity	Definition
*Training*	Instruction of MOH personnel to carry out the administrative and functional activities of the MDA and instruction of volunteers to develop skills required for the MDA.
*Mapping*	Testing to establish microfilaria and/or antigenaemia prevalence in communities.
*Mobilization and Education*	Media campaigns and community activities to increase MDA participation.
*Drug Distribution*	Logistic aspects of management of the drugs as well as administration of the drugs to the population.
*Adverse Reaction Monitoring*	Observation and treatment of persons suffering adverse reactions due to the MDA.
*Surveillance and Laboratory*	Tracking of community members in MDA area, laboratory work for testing, case identification, etc.
*Administration*	Supervisory work and paperwork to support the MDA.
*LF Non-MDA Costs*	Costs related to LF, but not the MDA (excluded from cost calculations).
*Other Non-LF, Non-MDA Costs*	MOH costs not at all related to the MDA or to LF (excluded from cost calculations).

### Study Sites

Study sites were chosen purposively to be representative of the LF- endemic regions in the countries (Table 3). National estimates were developed using the data from these representative study sites.

**Table 3:** Country Background Information.

Country	Total Population (millions)^a^	Estimated Population At-Risk for LF (millions)	Drugs Used	Areas Included in Cost Analysis	Drug Distribution Strategy
Burkina Faso	12.62	12.62	Mectizan albendazole	2001: Gaoua Region	NPELF is integrated in the health system. House-to-house by volunteers
				2002: Gaoua, Tenkodogo, Koudougou (2 of 5 districts) and Manga Regions	
Dominican Republic	8.62	1.5	DEC albendazole	2002–2003: Southwest area	Year 1: community distribution (house to house)
					Year 2: partial integration with primary health care system (house to house)
Egypt	70.51	2.57	DEC albendazole	2000: Qalyubia, Menofia, Sharkia, K. El Sheikh, Dakahlia, Gharbia, Giza, Governorates	House-to-house by teams of doctors and nurses
				2001: same plus Assiut Governorate	
Ghana	20.47	6.02	Mectizan	2003: Ahanta West, Builsa and Lawra Districts	Distribution posts, house-to-house or combination of two by volunteers
			albendazole		
Haiti	8.22	6	DEC	2000–2003: Leogane	Distribution posts by program staff
			albendazole	2002: Milot	
Philippines	78.58	23.5	DEC	2003: Sorsogon Province	Distribution posts, house-to-house by volunteers
			albendazole		
Tanzania	35.23	31.17	Mectizan	2000: Mafia District	First at distribution posts, then house-to-house follow-up by health care workers and volunteers
			albendazole	2001: Mafia and Mkuranga Districts	
				2002: Mafia, Mkuranga, Masasi Districts	
				2003: Mafia, Mkuranga, Masasi and Kilwa Districts	

a (World Health Organization 2002, World Health Organization 2003, World Health Organization 2004a).

Tanzanian investigators studied four districts located on the eastern coast, Kilwa, Mafia, Mkuranga and Masasi for the years 2000–2003. Data collection for the first three years was retrospective.

Data collected retrospectively in Burkina Faso documented the first two years of the MDA in the region of Gaoua in the first year and expansion into Tenkodogo in the second year.

The Philippines team chose to study seven municipalities and one city in the province of Sorsogon. These were selected based on filariasis endemicity using microfilaria rates, accessibility and population size. Half had high microfilaria rates (MF), half were easily accessible, while one quarter had large populations. In each municipality, one sentinel barangay or village was selected based on high MF rates and one to three adjacent barangays were also studied.

Cost data collection in Haiti was conducted in Leogane, where the first MDAs took place. Located 30 km west of Port-au-Prince in the south, Leogane is one of the highest risk areas in the country. Except for lower coverage in year-2 (2001) of the MDA program (because of side reactions that occurred after the first MDA) the program expanded not only in Leogane in 2002 but also to Milot, another high risk area just south of Cap Hatien in the north.

Researchers in the Dominican Republic collected cost data for the first two MDA campaigns. These were launched in Barahona on the southwest coast of the country.

The Ghana study costed three districts from the two epidemiological zones in the country, north and south. The districts were selected to reflect differing levels of program assistance. Builsa in the upper eastern region of the country received government support only, while Lawra in the upper west and Ahanta West on the west coast also received NGO support.

The cost analysis in Egypt covered the eight governorates along the Nile affected by LF. Costs were obtained for one district in each governorate and applied to the rest of the affected areas in the governorate based upon information about numbers of persons at risk, participating government personnel and quantities of medication distributed.[16]

### Data Collection

Data collection was both retrospective and prospective. The number of rounds of MDA costed per country ranged from one to four, with most countries costing two rounds. Countries developed national estimates for the program using data collected from a sampling of sites representative of the program (Table 3). Coverage rates are defined as the number of individuals reported to have ingested the antifilarial drugs divided by the total at-risk population in the program area. Those excluded from treatment included pregnant women, lactating women in the 1^st^ week post-partum, the very sick, children under two years of age in countries where DEC plus albendazole is the MDA regimen, and children under 90 cm in height (generally under 5 years of age) where albendazole is administered with Mectizan.

The data were collected from national, regional and district levels of the health care system via pre-tested questionnaires and spreadsheets, sometimes with the assistance of other agencies such as Ministries of Agriculture and Information. As LF is but one of many population-based health programs, most inputs (including personnel time, facilities, equipment, supplies, vehicles and fuel) were often shared by more than one program, and costs were apportioned accordingly. To capture the actual costs and percentage of the resources dedicated to LF, the teams reviewed program records and interviewed LF program administrators and personnel about allocations of personnel and resource time per year. Government tax fees such as customs tax on the drugs and the road tax were excluded.

## Results

### Country MDA Costs

As indicated in Table 4, the Financial costs per person treated ranged from $0.06 to $2.23 while Economic costs varied between $0.40 and $5.87. MDA coverage rates in the study populations ranged from 53% to 91%. While cost per person at risk can be easily calculated, cost per person treated is the more useful summary of costs for the purposes of planning and operations.

**Table 4:** Financial and Economic per Person Treated.

Country	Year	MDA round	Population at Risk in Current MDA Areas	Population Treated	Financial Cost ($US)	Financial per Person Treated ($US)	Economic Cost per Person Treated ($US)	MDA Coverage Rate (%)
*Albendazole+Mectizan*								
Burkina Faso	2001	1	559,000	431,399	$46,000	0.11	4.55	77%
Burkina Faso	2002	2	2,613,000	1,801,125	$110,000	0.06	4.82	69%
Ghana	2002	2	1,650,000	1,223,122	$1,358,000	0.17	4.88	69%
Tanzania	2000	1	40,800	37,000	$20,000	0.54	5.16	91%
Tanzania	2001	2	182,000	118,220	$50,000	0.42	5.82	65%
Tanzania	2002	3	537,000	437,698	$118,000	0.27	4.56	82%
Tanzania	2003	4	687,000	511,671	$133,000	0.26	4.53	75%
*Albendazole+DEC*								
Dominican Republic	2002	1	142,000	115,411	$216,000	1.87	3.10	83%
Dominican Republic	2003	2	333,000	250,059	$217,000*	0.87*	1.56*	75%
Egypt	2000	1	2,088,000	1,795,553	$2,412,000	1.37	1.80	86%
Egypt	2001	2	2,638,000	2,320,602	$3,109,000	1.00	1.34	87%
Haiti (Leogane)	2000	1	150,000	105,750	$236,000	2.23	n/a	71%
Haiti (Leogane)	2001	2	150,000	79,713	$156,000	1.96	n/a	53%
Haiti (Leogane)	2002	3	150,000	121,139	$158,000	1.30	n/a	81%
Haiti (Milot)	2002	1	126,000	100,376	$110,000	1.10	n/a	79%
Philippines	2003	3	691,000	556,912	$105,842	0.19	0.40	81%

* Adjusted for peso devaluation.

**Figure 1:**
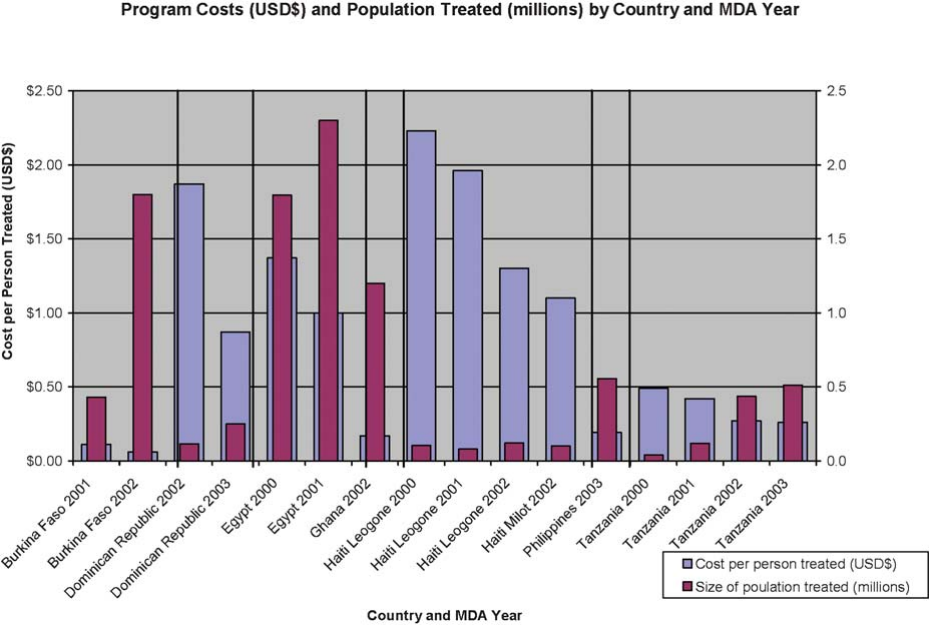
Financial Costs and Population Treated by Country and MDA Year.

Of the several trends that can be seen in Table 4, the most notable is that for those countries for which there is more than one year of data, cost per person treated decreased after the first year of the program, especially as the number of persons treated increased (see Figure 1). In addition, the Financial costs per person treated for Burkina Faso, Ghana, Tanzania and the Philippines, all of which used volunteers, were the lowest among the seven countries participating in the study.

### Activities and Inputs

Identification of resource allocation by activity and input is a useful and informative outcome of this study.

The use of resources for different activities varied among countries, and Table 5 identifies the proportion of the average national Financial costs expended for each activity in a ‘non-start-up’ MDA round (since yearly costs tend to stabilize after the ‘start-up’ year). Drug distribution generally represented the largest proportion of financial expenditures (average of 46%), with social mobilization/education and administration being next most prominent.

**Table 5:** Costs by Activity – Percentage of Financial Costs for a ‘Non-Start-Up’ MDA Round.

National Program	MDA Round	Training	Mapping^1^	Mobilization/Education	Drug Distribution	Adverse Reaction Monitoring	Surveillance/Lab	Admin.
*Albendazole+Mectizan*								
Burkina Faso	2	9%	0%	7%	45%	1%	5%	33%
Ghana	2	11%	2%	30%	13%	1%	27	16%
Tanzania^2^	4	20%	<1%	28%	40%	0%	3%	8%
*Albendazole+DEC*								
Dominican^3 ^Republic	2		0%	5%	89%		2%	4%
Egypt^4^	2	4%	11%	2%	47%	5%	12%	19%
Haiti^3^	2		5%	25%	49%	4%	12%	4%
Philippines	3	10%	10%	15%	38%	6%	3%	18%
AVERAGE		10.8	4.1	16.0	45.9	2.4	9.1	14.6

1 Major expenditures for mapping activities predated the years covered by the cost studies.

2 Average of district and central levels.

3 Haiti and the Dominican Republic distributed training costs across the activities.

4 Governorate level.

Analysis of financial costs by input, particularly useful for projecting budgets and for gauging the need for additional program support, again gave results varying appreciatively by country (Table 6). In all but one country, Egypt, the input that consumed the largest proportion of financial resources was personnel, averaging 53%, followed by supplies, equipment/facilities and transportation.

**Table 6:** Costs by Input – Percentage of Financial Costs for a ‘Non-Start-Up’ MDA Round.

National Program	MDA Round	Equip/Facilities	Personnel	Supplies	Transportation
*Albendazole+Mectizan*					
Burkina Faso	2	9%	47%	36%	8%
Ghana	2	21%	59%	1%	19%
Tanzania	4	24%	45%	17%	14%
*Albendazole+DEC*					
Dominican Republic	2	2%	56%	33%	8%
Egypt	2	16%	30%	53%	1%
Haiti	2	15%	61%	10%	14%
Philippines	3	5%	75%	18%	3%
AVERAGE		13.1	53	24	9.6

### Funding Sources

**Figure 2:**
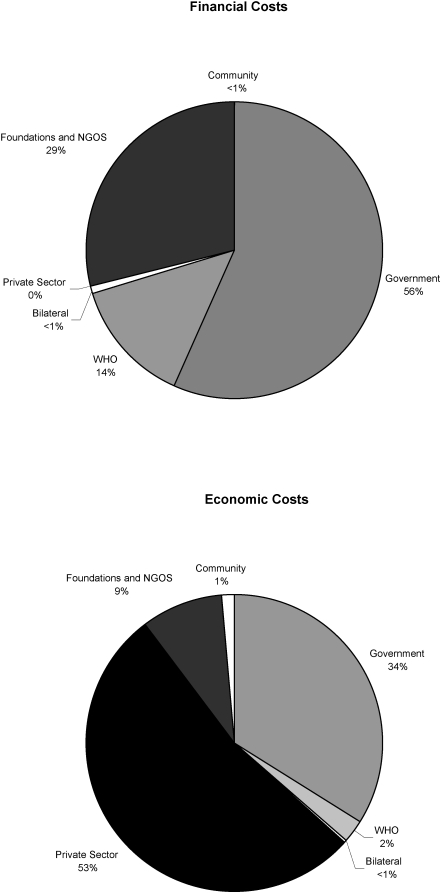
Sources of Funding for the MDAs Conducted by National LF Elimination Programs in Burkina Faso, Dominican Republic, Egypt, the Philippines and Tanzania (Financial Costs and Economic Costs).

These cost analyses not only identified how program resources were allocated, but data also were used to identify sources and amounts of funding for 5 of the 7 study countries. Funding was categorized as coming from national governments (excluding external donations for LF), international organizations (IDAs, NGOs, WHO), pharmaceutical companies and local communities (Table 7). As expected, the drug donations represent a large proportion of contributions to MDA programs, so that when MOH and partner contributions are examined from the perspective of *Economic costs*, the drug donations can make up over 90% (range 9%–99%) of the costs. This was particularly true in countries like Burkina Faso and Tanzania where both drugs used in the MDA (albendazole and Mectizan) were donated. When *Financial costs* are analyzed, however, it is clear that contributions from national governments represent a significant portion of the resources used to implement the MDAs (average = 56%, range 9%–99%). These relationships can be seen graphically in Figure 2 which presents the combined average of Financial and Economic funding sources for the Burkina Faso, the Dominican Republic, Egypt, the Philippines and Tanzania programs detailed in Table 7.

**Table 7:** Sources of Funding for LF Elimination Costs.

National Program	Year	National Government	International Organizations (IDA,NGO,WHO)	Pharma Companies		Community
*Percentage of* Program *Costs (*i.e., *Financial Costs) for Each Source of Funds*						
*Albendazole+Mectizan*						
Burkina Faso	2002	39%	61%	0%		<1%
Tanzania	2003	55%	45%	0%		<1%
*Albendazole+DEC*						
Dominican Republic	2003	9%	91%	0%		<1%
Egypt	2000	80%	20%	0%		<1%
Philippines	2003	99%	<1%	0%		<1%
*Percentage of* Total *Costs (*i.e., *Economic Costs) for Each Source of Funds*						
*Albendazole+Mectizan*						
Burkina Faso	2002	<1%	<1%	99%		<1%
Tanzania	2003	3%	2%	95%		<1%
*Albendazole+DEC*						
Dominican Republic	1998–2003	46%	42%	9%		3%
Egypt	2000	72.5%	11.5%	16%		<1%
Philippines	2003	48%	3%	46%		3%

### Sensitivity Analyses

Sensitivity analyses were conducted on the personnel input (data not shown) to gauge how much an increase in personnel costs would impact overall costs. Personnel was selected for sensitivity analysis because, with the exception of Egypt, this input represented the largest source of costs in the participating countries. Sensitivity analyses conducted on personnel input for Ghana and Burkina Faso demonstrated that increasing the proportion of personnel time dedicated to the LF MDA did not raise financial costs beyond the currently-calculated ranges, even under conditions where personnel time was doubled (Ghana - US$0.25 and Burkina - US$0.08) or tripled (Ghana - US$0.34 and Burkina - US$0.11). Additionally, a sensitivity analysis of personnel costs in the Philippines showed that personnel costs would remain well within the range of the current Financial costs (US$ 0.69) even if personnel time were doubled.

### Study Limitations

There were three principal study limitations. First, investigators frequently encountered problems in estimating the proportion of time and money allocated specifically to the LF MDA programs. Staff had not previously tracked time apportioned to LF-specific activities, and since LF programs are small in relation to other MOH initiatives (consuming anywhere from 1% to 5% a year in most countries), recall was problematic at times. Similar situations were encountered with respect to other resources such as transportation, facilities and equipment dedicated to LF. Second, interpretations of the definitions of certain activity categories sometimes differed among researchers applying the cost analysis protocol and data collection instrument. At times questions arose about allocation to one or the other of categories mentioned. Third, while study teams attempted to identify the original source of funds, the original funder may not have been correctly identified in all cases, especially where money was channeled through one or more intermediary sources.

## Discussion

Good cost analysis is essential to provide an evidence-based rationale for investing in LF elimination. The key target figure is the cost per person treated. Few results have been available to date, [12,16] and little has been documented about the principal determinants of cost per person treated. This study has defined key activities and input categories and gathered cost information via the standardized cost-analysis studies of seven national LF MDA programs.

One of the most striking findings of the present studies is the variability in the Financial cost per person treated. Differences in programmatic structure can account for some of these differences because each country tailors the LF elimination strategy for a best fit to local conditions. But beyond the program-specific differences, the principal determinants underlying the variability appeared to be: 1) the newness of the country program (*i.e.,* the number of years it had been running), 2) the use of volunteers, and 3) the size of the population treated.

### Newness or Round of MDA

MDA program start-up years resulted in higher Financial and Economic costs per person treated than did subsequent years. Inevitably, there are more costs and reallocation of resources in the first year of a program. At the same time, new programs typically cover a limited geographic area and a relatively small population in their first year. As the population covered expands after the first year, the cost per person treated tends to drop.

In addition to extra expenditures in a start-up year as compared to other years, costs can also decrease over time because new cost-savings strategies are identified and implemented. For example, in the first MDA year in the Dominican Republic, health workers from non-governmental organizations participated in the MDA; these individuals were paid per diem for all of the time they participated, in some cases higher than the wages of workers in the health system. MDA integration into the health system contributed to the 50% decrease in the second year Program cost per person treated. Similarly in Haiti, after the first year cost analysis revealed that 22% of resources were dedicated to adverse reactions, the adverse reaction protocol was revised to an equally effective but less costly strategy. In the second year only 4% of resources were used for adverse reactions.

### Use of Volunteers

The use of volunteers had the greatest impact on costs. In Burkina Faso, Tanzania, Ghana, and the Philippines - where Program costs are lowest - health workers are employed down to the sub-district level, and volunteers, who are compensated very little in the LF program, work at the village level. Volunteers in these countries contribute a large proportion of the time dedicated to the MDA (frequently receiving per diem only for days in training) and at times provide their own transportation (e.g., Burkina Faso). However, while there is a strong relationship between the use of volunteers and lower cost per person treated, this does not suggest that any country choosing to use volunteers would see savings of 85%. Country-specific conditions that lead to the use of volunteers may also determine lower costs overall.

The use of volunteers leads to the question of how to accurately value the time volunteers ‘donated’ to each program. One traditional approach to valuing volunteer time is to apply the wage from the volunteer's normal paid employment and value their volunteer time accordingly. The problem with this, as pointed out by McFarland *et al*.[18] in a report on the costs of onchocerciasis MDA, is that many volunteers are subsistence farmers who do not participate in the formal labor market. One alternative is to decide the fair market value of the time, i.e. the amount the volunteer would be paid if the program had to hire individuals for the work,[19] or another, by using an estimate from prior studies in a similar setting.[18]

In the country programs included in this study, a diverse group of individuals, comprising students, teachers, farm laborers, and elderly retirees, served as volunteers. Owing to this fact and the economic conditions in the participating countries, applying traditional methodologies for costing volunteer time may not be appropriate. After several countries explored volunteer participation in MDAs and the earning capacity of volunteers in their regular pursuits, a decision was made by the investigators in all participating countries not to include these costs. Therefore, while the study included the per diem paid to volunteers during training, it did not account for the opportunity cost of the volunteer's time dedicated to the MDA itself. The evaluation of the community contribution from countries which used volunteers is very definitely underestimated.

Program managers can control, to some extent, the use of volunteers and might explore this strategy in resource-constrained environments. Beyond the monetary savings, there is a benefit from connecting the program with volunteers who are opinion leaders from different community sectors. So while initially decisions to utilize volunteers might be financially based, countries ultimately can benefit not only from volunteers' labor but also from their connections to the communities in which they serve. However, program managers must bear in mind the competition for volunteers from other health programs which sometimes pay volunteers more for their efforts. The sensitivity analyses on personnel time devoted to the MDA point to the possibility that it may be worthwhile exploring opportunities to increase remuneration for volunteers. Indeed, both valuing and best utilizing volunteer time merit continued examination.

### Size of Population Treated

The third principal source of variation in cost per person treated was the size of the population treated, an element that can be controlled by program managers during program expansion. When programs scale-up, the cost per person treated drops, primarily because most of the overhead costs are associated with start-up costs at the national and district levels. Once the system has been established, the majority of the new costs are in the new area (district or governorate) that is being covered. In Burkina Faso and Tanzania the Financial costs per person were halved as the MDA covered larger populations. Between 2001 and 2002 Program costs in Burkina declined 45% while the population treated increased by over 400%. The progression in Tanzania between 2000 and 2003 was similar, with the Financial cost per person treated decreasing by 47% while the treated population increased over 13-fold. Between 2000 and 2001, the number of persons treated in Egypt increased 29%, while the Financial cost per person treated decreased 27%.

Such findings emphasize the need to keep current programs adequately funded so that these programs can expand and increase the number of persons treated and thereby capture the savings resulting from the economies of scale.

Once the principal determinants of the cost per person treated are identified, they can be used to manage program costs either by making internal changes within a program, such as scaling-up and increasing efficiency, or by taking advantage of existing external resources, as through integration. LF elimination programs can be integrated into the existing health system, as in the Dominican Republic, or with other preventive chemotherapy programs. For the LF elimination program, costs per person treated are within the range of those estimated for other similar disease control and elimination programs; namely soil-transmitted helminths ($0.25 per treatment[20]), trachoma ($0.50 per treatment[21]) and onchocerciasis ($0.58 per treatment[18]). The potential programmatic overlap of activities among these and other public health initiatives includes administration, drug distribution, monitoring, surveillance, and social mobilization, so attractive cost-saving opportunities can be envisioned through integration. While estimates place the cost savings from integrated delivery between 25% and 47%,[22,23] the LF cost analysis protocol utilized in the present cost study can provide a useful tool by which to document the potential savings from integrating some or all programmatic activities of these initiatives. Indeed, countries that have already completed the LF cost analysis are well placed to estimate costs of integrated programs, given that many of the costs common to all programs have already been identified.

Also of particular interest from this study was the documentation of the substantial contributions (*i.e.,* program *ownership*) by national governments (Figure 2). On average 56% of Financial costs of the LF elimination programs were financed by governments. The Egyptian government contributed 80% of Program costs, including participation of the Ministries of Health and Population, of Agriculture, and of Information. This is consistent with similar programs for TB and malaria which estimate that 70% of Financial costs are paid for by national governments. [24,25] Furthermore, LF MDA programs do not require new or additional funding for all inputs. For example, the portion of the salary of a District Medical Officer (DMO) spent on the project would be included in the analysis as a program cost, but the DMO's salary would have been funded regardless of whether the time was spent on LF MDA activities or other programs and may not require additional financial outlays. Governments have a choice as to where they use their resources, and the percentages noted in this study emphasize the commitment these national governments have made to LF elimination.

### Resource allocation choices

Surveillance and mapping activities represented a significantly higher proportion of resource requirements in Egypt than in other countries (between 10% and 12% at both the national and governorate levels), since Egypt has selected small MDA implementation units, i.e. the village level, and hence focused on monitoring potential ‘at-risk’ areas closely in a large number of implementation units.

Tanzania's national program chose to invest heavily in social mobilization and education to assist the districts in raising awareness in the general population for current and future MDAs. The districts placed more emphasis on funding drug distribution and personnel training.

### Conclusion

The principal aim of this study was to provide critical information regarding the cost of implementing MDA for the prevention of lymphatic filariasis. The study demonstrates that the costs of MDA programs for LF elimination are comparable to those estimated for other similar disease control and elimination programs. While some programs had costs per person treated of over a dollar, it was quite straightforward to identify those factors most affecting program costs.

Such findings can be used on a national scale for program planning, development and fundraising, and on a global scale for calculating current global costs, predicting scale-up costs and calculating savings from integration with other programs. These results also will form the basis for guiding cost-effectiveness and cost-benefit analyses, as more information on the effectiveness of MDAs in the study countries becomes available. Additionally, the analytic tool used in this study will be valuable for further studies of the LF elimination program including costing of LF disability alleviation activities and the process certifying LF elimination.

A further finding of particular importance was documentation of the impact that the use of volunteers has on program costs. Further research on how best to utilize volunteerism for such public health programs could contribute appreciably to ensuring success of the MDA-based programs and to anchoring them in the communities they seek to protect.

Finally, implementation of this study produced a number of ancillary program benefits. The process of defining and reviewing costs allowed for a review of operations at all levels (inputs, processes and outputs) that was also frequently used to assess efficiency. The findings provide the opportunity for the development of cost-effective implementation models built on best practices from each country; hopefully, these can be adopted and adapted by new programs from the start, particularly in Africa where almost 30 more endemic countries still need to initiate LF elimination programs.
